# Supplementary material for: Transcriptomic Insights into Mechanisms of Early Seed Maturation in the Garden Pea (Pisum sativum L.)
Source: Cells. 2020 Mar 23;9(3):779. doi: 10.3390/cells9030779 (PMC7140803; doi:10.3390/cells9030779)

Sprint−2(10)

Global Summary

%DE = 0.91

genes with fdr < 0.2 = 1901 (783 + / 1118 −)

genes with fdr < 0.1 = 1837 (744 + / 1093 −)

genes with fdr < 0.05 = 1701 (666 + / 1035 −)

genes with fdr < 0.01 = 1481 (539 + / 942 −)

genes in genesets = 0

<FC> = 0

<t−score> = −1087.54

<p−value> = 0

<fdr> = 0.09

Global Genelist

Rank	ID	log(FC)	p−value	fdr	Metagene	Description
1	comp30001_c	0	0e+00	2e−13	18 x 14	
2	TRINITY_DN3	−0.19	1e−15	2e−11	4 x 30	
3	TRINITY_DN5	−0.88	1e−13	4e−11	1 x 30	
4	TRINITY_DN1	−4.78	3e−13	8e−11	1 x 1	
5	TRINITY_DN4	−5.76	7e−13	9e−11	1 x 30	
6	TRINITY_DN4	−4.32	1e−12	9e−11	1 x 30	
7	TRINITY_DN8	−0.26	2e−12	5e−10	25 x 30	
8	TRINITY_DN1	−0.2	5e−12	5e−10	4 x 27	
9	TRINITY_DN5	−2.06	7e−12	6e−10	1 x 30	
10	TRINITY_DN4	−0.47	1e−11	5e−09	29 x 30	
11	TRINITY_DN5	−0.28	5e−11	5e−09	3 x 1	
12	TRINITY_DN4	−0.31	6e−11	5e−09	1 x 28	
13	TRINITY_DN1	−0.53	8e−11	7e−09	1 x 1	
14	TRINITY_DN3	−0.02	1e−10	7e−09	13 x 22	
15	TRINITY_DN3	−0.48	2e−10	7e−09	1 x 1	
16	TRINITY_DN2	−0.56	2e−10	7e−09	1 x 1	
17	TRINITY_DN2	−0.18	3e−10	7e−09	23 x 30	
18	TRINITY_DN3	−0.17	3e−10	7e−09	1 x 25	
19	TRINITY_DN2	−0.05	3e−10	1e−08	16 x 26	
20	comp43362_c	−0.07	4e−10	1e−08	7 x 4	

Portrait

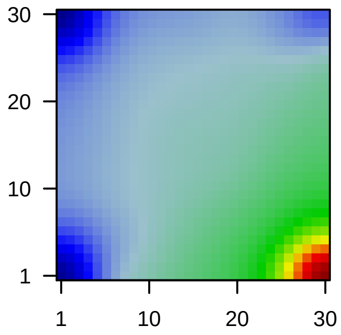

Top 100 DE genes

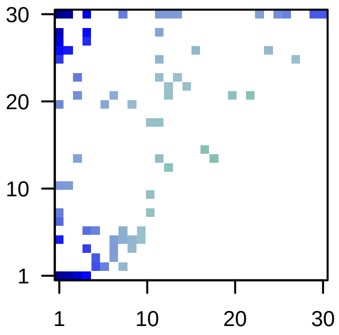

p−values

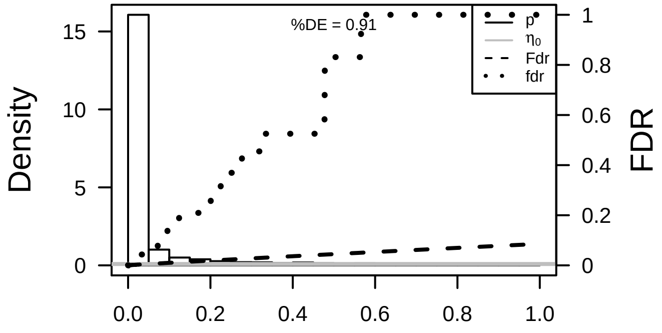

Supplement: Supplementary file 1 [file cells-09-00779-s001.zip › Supplementary materials/FileS4.zip!/FileS3/TFs - Results/Summary Sheets - Groups/Reports/Sprint.2.10..pdf]
